# Interaction of CD200 Overexpression on Tumor Cells with CD200R1 Overexpression on Stromal Cells: An Escape from the Host Immune Response in Rectal Cancer Patients

**DOI:** 10.1155/2019/5689464

**Published:** 2019-01-21

**Authors:** Atil Bisgin, Wen-Jian Meng, Gunnar Adell, Xiao-Feng Sun

**Affiliations:** ^1^Department of Oncology and Department of Clinical and Experimental Medicine, Linköping University, Linköping, Sweden; ^2^Medical Genetics Department of Balcali Clinics and Hospital, Faculty of Medicine, Cukurova University, Adana, Turkey

## Abstract

CD200 imparts an immunoregulatory signal through its receptor, CD200R1, leading to the suppression of tumor specific immunity. The mechanism of CD200:CD200R1 signaling pathway is still uncertain. Our aim was to investigate the expression and localization of CD200 and its receptor CD200R1 and their clinical significance in rectal cancer patients. We examined the immunohistochemical expressions and localizations of CD200 and CD200R1 in 140 rectal cancer patients. Among the patients, 79 underwent the preoperative radiotherapy and the others were untreated prior to the surgery. In addition, 121 matched normal rectal mucosa samples were evaluated. The results of immunohistochemical analysis showed a strikingly high level of CD200 in tumor cells (p=0.001) and CD200R1 expression in normal mucosal epithelium and stromal cells. Importantly, CD200R1 was overexpressed in stromal cells of the metastatic cancer patients compared to patients without metastases (p=0.002). More than that, 87% of metastatic patients had a phenotype of upregulated CD200 in tumor cells accompanied by overexpressed CD200R1 in stromal cells. In addition, low levels of CD200 were correlated with improved overall survival in untreated patients. We showed that tumor-stroma communication through CD200 and its receptor interaction is selected in patients with high risk of relapse. High levels of these molecules support instigation of the far and local metastatic nest that provides solid ground for metastasis. Our current data also disclose a mechanism by which CD200:CD200R1 affects tumor progression and may strengthen the feasibility of targeting CD200 or CD200R1 as anticancer strategy.

## 1. Introduction

Cancer progression is a multistep process including the most critical steps; tumor invasion and metastasis that are the major causes of cancer deaths and the obstacles to the successful treatment [1]. Many studies focused on identifying the progression and metastasis controlling genes [2, 3]. However, cancer metastasis is also dependent on the stromal compartment not exclusively regulated by intrinsic genes in cancer cells. Many studies have shown the interaction of cancer cells and stromal cells. CD200:CD200R1 signaling is one of the pivotal members of inflammatory signaling that has been shown recently [4].

CD200 is a membrane glycoprotein that widely expressed multiple cells/tissues [5, 6]. Its distinctive expression was subsequently studied by a number of different groups, confirming that high expression of CD200 was an independent prognostic factor and predicting reduced overall survival in a number of hematological malignancies, including multiple myeloma, acute myeloid leukemia (AML), and chronic lymphocytic leukemia (CLL) [7–9]. So the question has arisen, given this role as a prognostic factor in human blood malignancies, as how CD200 is involved in cancer. A hypothetical model suggested that the overexpression of CD200 is associated with inhibition of tumor-specific immunity by switching the cytokine profiles from T-helper 1 cells (Th1) to T-helper 2 cells. There are also most recent studies showing that CD200 expression controls two other pathways; one is a regulatory T cell expansion and disease progression in acute myeloid leukemia (AML) and chronic lymphocytic leukemia (CLL); other one is the checkpoint blockade that augments cytotoxicity of cytokine-induced killer cells against human myeloid leukemia blasts [10, 11]. This mechanism may take part in loss of antitumor control and results in immunocompromised tumor environment [8, 12].

In vitro and in vivo studies showed that CD200-expressing tumor cells can suppress T-cell responses [8, 12, 13]. On the other hand, CD200 is also a ligand for a structurally similar receptor CD200R1 that imparts an immunoregulatory signal [14]. This interaction is one of the most important immunological checkpoints: leading to the suppression immune responses [15]. The most recent in vitro studies suggest that blocking this CD200:CD200R1 interaction enhances Th1 responses and that is how the CD200-expressing cancer cells survive immune therapy or a natural immune response [8, 16, 17].

As a member of immune inhibitory receptors CD200R1 has another important role for the maintenance of immune tolerance and its expression is more restricted on myeloid and lymphoid lineages of cells. Tumor cells mostly use these immune inhibitory receptors for their benefits. Through this endogenous inhibitory pathway, CD200:CD200R1 interaction may also be featured in tumor progression, outgrowth, and/or metastasis. This idea was confirmed in one member of epithelial tumors, in skin carcinogenesis [18–20].

We set our study on molecular screening of tissue samples from rectal cancer patients. In this scenario, we investigated the expression and localization of CD200 and its receptor CD200R1 to identify a molecular marker useful for determining prognosis through routine clinical assessment with clinicopathological findings.

## 2. Material and Methods

### 2.1. Clinical Assessment of Rectal Cancer Patients

Paraffin-embedded tissues from rectal cancer patients were evaluated for our study. The study was approved by the local committee on ethics. Demographic and clinical data of the patients are given in Table 1.

There were two rectal cancer patients' groups: first group underwent the preoperative radiotherapy treatment as named “RT group” and second which is the patients without any treatment prior to the surgery was named “untreated group”. The mean age at the time of diagnosis was 62.6 years in RT group and 63.4 in untreated group. All patients were pathologically staged according to the American Joint Committee on Cancer, also known as the TNM system. Most individuals had earlier stages: 56 of Stages I, II, and III (91%) and 5 cases of Stage IV (9%) in RT group and 74 cases of Stages I, II, and III (94%) and 5 cases of Stage IV (6%) in untreated group. Patients were also categorized by the differentiation grade from poor to good. Using this grading system by pathologists; in RT Group 20 cases had poor differentiation (33%), 36 cases had moderate differentiation (59%), and 5 cases had good differentiation (11%), while 16 patients with poor differentiation, 59 with moderate differentiation, and 4 with good differentiation were classified in untreated group.

Additionally, since the proliferation of cancer cells is thought to be a key feature of progression, Ki-67 expression pattern was added into the clinicopathological findings to assess the proliferative activity that had been previously reported by our group [21].

### 2.2. Immunohistochemistry

All primary antibodies used for CD200:CD200R1 immunohistochemistry were obtained from AbCam (anti-CD200/OX2 antibody [OX-104], ab33363; anti-CD200R antibody [OX-108], ab17225). Since CD200 and its receptor CD200R1 were predominantly expressed on myeloid and lymphoid cells, samples of lymphoid tissues and lymph node sections were included as positive controls to optimize the primary antibody titers. Negative controls were stained only with the appropriate secondary antibody.

Immunohistochemical analysis was carried out according to the antibody staining protocol in tissue microarray slides (TMAs). Benign and malignant tissues were stained using the above-stated primary antibodies. The slides were counterstained with hematoxylin and then coverslipped.

### 2.3. Analysis of Immunohistochemical Staining for CD200 and CD200R1 Expressions

Immunohistochemical analysis of TMAs was performed by 2 independent, dedicated clinicians without any prior knowledge of the paired specimens and clinical data. Sections from normal rectal mucosal samples and primary tumor samples that were prepared from the resection of the tumor by surgery were analyzed. Primary tumor samples were assessed into two regions and analyzed separately for the tumoral and stromal area around the tumor. Both intensity and marker distribution (percentage of the positively stained cells) were used for the semiquantitative scoring. Intensity was scored as 0 for negative, 1 for weak, 2 for moderate, and 3 for strong. The distribution was also similarly scored as follows: 0 ≤ 10%; 1 = 10% to 45%; 2 = 46% to 70%; 3 = 71% to 85%; and 4 ≥ 85%. The final immunostaining score was then calculated by adding both intensity and marker distribution scores.

### 2.4. Statistical Analysis

Statistica v.10 software for Windows was used for the statistical analyses. Statistical differences were evaluated when the probability level is less than 5% (p<0.05). The standard errors of the means are shown as error bars in the figures. Since the normal distribution was not detected by Kolmogorov-Smirnov test, comparison of each two groups was conducted by Mann-Whitney* U *tests. Spearman Rho analyses were revealed for the correlation analysis between CD200:CD200R1 profile and clinical findings.

## 3. Results and Discussion

### 3.1. Expressions of CD200:CD200R1 for Immunohistochemical Analysis and Confirmation for Scoring of Rectal Cancer Specimens

Optimization of the primary antibody concentrations of CD200 and its receptor CD200R1 was performed using lymph node sections regardless of disease before the analyses. While primary antibodies generated a well staining pattern on node sections in 1:200 dilution for CD200 and 1:50 dilution for CD200R1, lymph node sections that were treated with the secondary antibody alone as a negative control did not yield any staining (data not shown). These results support that the CD200 and CD200R1 expression on paraffin-embedded rectal cancer tissue samples could be detected by immunohistochemical staining protocol and then scored.

### 3.2. Rectal Cancer Tissues Displayed Increased CD200 Expression Compared with Normal Rectal Mucosa

**Figure 1:**
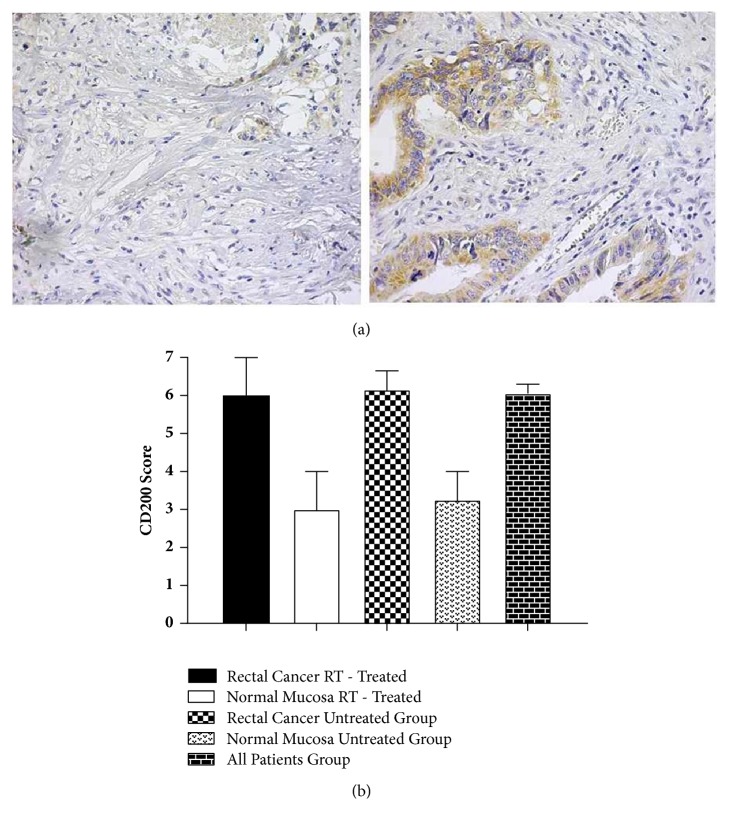
(a) Immunohistochemical staining of CD200 in total 140 rectal cancer patients. Representative images (magnification x 60) are provided from different patients with low (on the left) and high scorings (on the right). Brown precipitate indicates positive staining. (b) Semiquantitative scoring of CD200 immunohistochemical staining in patients with rectal cancer and normal mucosa. Scoring was performed as described in the section of Materials and Methods.

Total of 140 paraffin-embedded tissues of rectal cancer patients were analyzed for determining the CD200 and CD200R1 expression by immunohistochemistry. Seventy-nine of 140 are the samples from untreated group and 61 of 140 are from patients that underwent radiotherapy treatment prior to the surgery. In addition, 121 normal rectal epithelial mucosa samples from the distant area were stained and then scored. Fifty-five of 121 were from preoperative radiotherapy given patients and the rest were from the untreated group of patients. Representative immunohistochemical staining images are shown in Figure 1(a), and Figure 1(b) shows the comparison of the both normal mucosa and rectal cancer groups. In patients with rectal cancer in independence of the radiotherapy given or not, overexpression of CD200 was detected in contrast to normal mucosa samples (Suppl.  Table 1). The expression levels difference was statistically significant (p = 0.001).

### 3.3. Overexpression of CD200 Represents Similar Profiles to Those of Rectal Cancer Patients with or without Preoperative Radiotherapy

To compare CD200 and CD200R1 expression profiles of rectal cancer patients depending of the preoperative treatment modality, two groups; one underwent preoperative radiotherapy and the other without any treatment, were assessed. Patients displayed higher levels of CD200 and this high CD200 expression was similar in both groups (Figure 1(b)). The difference did not reveal any statistical difference (p = 0.597).

### 3.4. The Significance of CD200 Expression on Normal Mucosa in Connection with Survival

The main outcome of developing different treatment protocols and all is overall survival. Based on our long period followed-up rectal cancer patients' data, the low levels of CD200 expression in normal epithelium (score ≤ 3) related to long survival (more than 5 years) in untreated rectal patients group according to the implemented correlation and survival test (p = 0.020) (data not shown).

### 3.5. CD200R1 Expression Profiles in Rectal Cancer Patients

**Figure 2:**
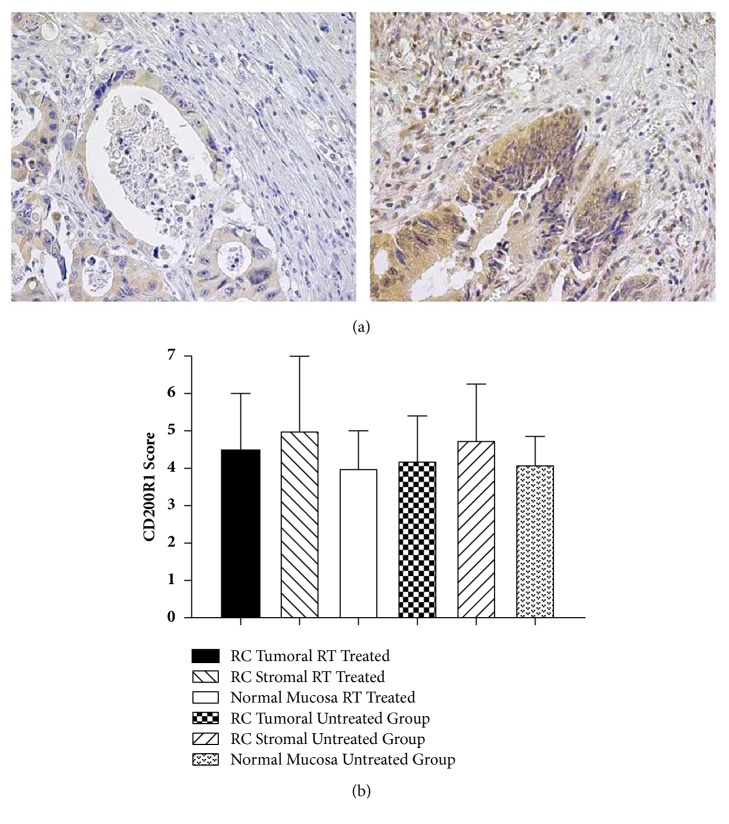
(a) Immunohıstochemical staining of CD200R1 in total 140 rectal cancer patients. Representative images including both the tumoral and stromal regions (magnification x 60) are provided from different patients with low and high scorings. Brown precipitate indicates positive staining. (b) Semiquantitative scoring of CD200R1 immunohistochemical staining in patients with rectal cancer tumoral expression and stromal expression and normal mucosa. Scoring was performed as described in the section of Materials and Methods.

Representative immunohistochemical staining for CD200R1 images of rectal cancer is shown in Figure 2(a), and Figure 2(b) shows the comparison of the all groups including normal mucosa and rectal cancer groups whom one group without any treatment and the other underwent preoperative radiotherapy. There was a distinctive CD200R1 expression profile, and no difference was noted between the groups after the statistical evaluation as follows; p = 0.434 in between normal mucosa and preoperative radiotherapy gorup; p = 0.482 in between normal mucosa and untreated group; and p = 0.570 in between two groups.

### 3.6. Overexpression of CD200R1 in Stromal Cells of Rectal Cancer Patients Correlated with the High Recurrence Risk and Metastasis

The main clinical parameter for the patients is the recurrence risk and metastasis. Based on this parameter, among all 140 patients 50 cases had metastasis and 34 cases had recurrences. In more details, 20 of 50 and 16 of 34 underwent preoperative radiotherapy and 30 of 50 and 18 of 34 were untreated rectal cancer patients. Interestingly, when we put all together, the high recurrence risk and metastasis group, CD200R1 was overexpressed in stromal cells of these patients compared to the no recurrence and nonmetastatic group. The correlation test was used to reveal any correlation. The overexpressed CD200R1 is correlated with high recurrence risk and the metastasis in rectal cancer patients in independent of preoperative radiotherapy (Suppl. Tables 2 and 3).

### 3.7. High Scores of CD200 in Tumor Cells Together with CD200R1 in Stromal Cells Related to Metastatic Pattern

Finally, we investigated evidence for a correlation between CD200:CD200R1 expression profile and the metastasis risk. Interestingly upregulated CD200 expression is when only associated with CD200R1 overexpression strongly correlated with metastatic pattern of rectal cancer patients (Suppl.  Table 4A). These high expression levels of over 6 in IHC scoring were shown in 87% rectal cancer patients with metastasis. A correlation between CD200 and its receptor CD200R1 expression was investigated using Spearman Rho Correlation analysis. This increased expression of CD200 on tumor cells was not correlated with CD200R1 expression on tumor or stromal cells (Suppl.  Table 4B).

### 3.8. Proliferative Activity of Rectal Cancer Patients

Proliferative activity was assessed in terms of the Ki-67 IHC staining in our groups' previous reported study [21]. Among the tissue sections analyzed, rectal cancer cells exhibited a wide range of Ki-67 expression that was ranged from 0 to 86 percent positivity, reflecting a variation in proliferative activity. However, neither clinical variables nor CD200:CD200R1 had any relation to Ki-67 expression.

## 4. Conclusions

Colorectal cancer is the second leading cause of cancer-related mortality and metastatic pattern still remains incurable [22]. Most recent studies have focused on the contribution of tumor microenvironment to the progression of tumors including colorectal cancer. A major contributor to the tumor microenvironment is inflammation and inflammatory mediators that are adept in supporting tumor cell growth, survival, and metastasis [23].

An important immunological checkpoint is CD200:CD200R1 signaling pathway that controls inflammation, immune tolerance, and antitumor immune responses [18]. Several studies in hematologic tumors suggested the CD200 overexpression as a prognostic factor, while some others showed the expression in solid malignancies [7, 9, 16, 19]. According to all published data, it has been proposed that CD200 expression plays a role in the ability to escape the immune system. More speculative most recently published findings were CD200:CD200R1 signaling suppressing antitumor responses and regulating the metastatic growth [20].

Tumor metastasis is a complex multistep process in which cell migration and invasion are important steps. Our most important finding was the correlation between metastatic pattern and high expression patterns of CD200 on tumor cells together with CD200R1 expression on stromal cells. These findings suggest a potential role of stromal cells and the interaction to the tumor cells. This cell-cell and ligand-receptor interaction might be one of the important steps of metastasis and oncogenesis.

Another most important fundamental in oncogenesis is the cellular proliferation, to maintain tissue homeostasis. For that reason, we assessed the relationship between expression patterns of tumor cell proliferation marker Ki-67 and CD200:CD200R1. There are some discrepancies in the literature that some reported no relation between Ki-67 immunoreactivity and various clinicopathological and prognostic variables in cases with colorectal carcinomas; on the other hand some of them reported its relation to histologic grade and pathological stage [21, 24, 25]. Still, Ki-67 is the most reliable and reproducible marker. In our study, there was no correlation between CD200:CD200R1 and Ki-67 but CD200:CD200R1 expression patterns were related to metastasis and recurrence. The results thus supporting the hypothesis that metastasis in rectal cancers has a linkage with host related stromal cells not only with tumor cells itself, neither may not regulated by only the proliferative activity that was assessed by Ki-67 staining.

On the basis of our findings, we propose not only the upregulation of CD200 on tumor cells, but also CD200R1 overexpression on stromal cells in terms of the interaction between CD200 and its receptor CD200R1, which are the hallmark of metastatic rectal cancer and potentially responsible for supporting the survival of CD200 expressing tumor cells.

CD200:CD200R1 interaction and the mechanism still remain significant unknowns. However, the immune inhibitory receptors/pathways already become important therapeutic targets to strengthen antitumor responses in cancer treatment during the last few years. Most recent studies confirmed the increase in chemotherapy activity within the synergy of CD200 (or CD200R) blockade to cure and to produce immune resistance to metastasis of metastatic breast cancer in mice models [11]. Moreover, there are on-going clinical trials against the inhibitory receptors including CTLA4, PD-1, and an antibody currently being evaluated where blocks CD200:CD200R1 (Alexion Pharmaceuticals, NCT00648739) have yielded promising results [26]. Our findings also suggest the extension of the therapeutic use of CD200:CD200R1 blockers to rectal cancer patients that might lead to the more effective treatment modalities.

Another revealed data of our study was CD200 expression on normal mucosa cells that had also an aspect of its relation to survival. However, this correlation is only limited in the untreated group. There might be 2 main explanations of this correlation in untreated group but not in preoperative radiotherapy patients. The fact that CD200 overexpression is a predictor of poor prognosis in a number of hematologic malignancies supports our data. Because of its immunosuppressive effect, low expression of CD200 attenuates the inflammation and the inflammation might enhance the survival of patients according to the antitumor effect. Second since the radiotherapy has an effect on normal tissue homeostasis, it may also affect the immune response, the repertoire, and future immune reactions to the tumor via the field effect.

Collectively, all these results highlight the strong contribution that tumor cells and stromal cells communication defined the metastatic outcome of rectal cancer patients. Particularly, our analysis implicates CD200 and CD200R1 have an effect on survival and metastasis. Because of the long follow-up time together with the different treatment modalities used and other clinical data in our large study group, we determined the correlations and clinical importance of CD200:CD200R1 receptor profile and their location of expression in rectal cancer patients.

In conclusion, we have identified CD200 and its receptor CD200R1 expression profiles and their location in tumor and tumor surrounding is, for the first time, demonstrated in rectal cancer patients. We have also combined the expression pattern in relation to clinical status and treatment. In summary, we showed that cross talk between tumor and stroma might support metastasis-specific functions. These results also point the CD200:CD200R1 expression profile might be useful to follow up rectal cancer progression by virtue of their connection to recurrence risk, metastasis, and survival depending on the treatment modality, whereas rectal cancer patients appear to be targets for adjuvant therapies directed at interrupting CD200:CD200R1 immunoregulatory axis.

## Figures and Tables

**Table 1:** 

**Characteristic**	**Rectal Cancer RT Group **	**Normal RT Group**	**Rectal Cancer Untreated**	**Normal Untreated **
	**n = 61 (**%**)**	**n = 55 (**%**)**	**n = 79 (**%**)**	**n = 66 (**%**)**
**Sex**				
**Male**	36 (59)	27 (49)	45 (56)	33 (50)
**Female**	25 (41)	28 (51)	34 (44)	33 (50)

**Age (years)**	62.6	61.7	63.4	62.7

**TNM**				
**I + II + III**	56 (91)		74 (94)	
**IV**	5 (9)		5 (6)	

**Differentiation Grade**				
**Good **	5 (8)		4 (5)	
**Moderate**	36 (59)		59 (75)	
**Poor**	20 (33)		16 (20)	

**Metastasis**				
**No**	41 (67)		49 (62)	
**Yes**	20 (33)		30 (38)	

**Recurrence**				
**No**	45 (74)		61 (77)	
**Yes**	16 (26)		18 (23)	

Patient characteristics. Rectal cancer RT group: rectal cancer patients underwent preoperative radiotherapy. Rectal cancer untreated: rectal cancer patients without any treatment prior to the operation. Normal RT group: normal rectal mucosa from rectal cancer RT group. Normal untreated: normal rectal mucosa from rectal cancer untreated group.

## Data Availability

The data used to support the findings of this study are included within the article.

## Supplementary Materials

Supplementary Materials Supplementary Table 1. Immunohistochemical scoring of CD200 in rectal cancer tissues compared to the benign epithelium (p=0.001). Supplementary Table 2. Comparative analysis of CD200R1 expression in cases with recurrence and without recurrence. Scoring was assessed on the stromal region of primary tumor samples. Supplementary Table 3. Comparative analysis of CD200R1 expression on stromal region of primary tumor sections. Cases compared between with or without lymph node metastasis. Supplementary Table 4. (A) The expression pattern of CD200 and CD200R1 in metastatic versus nonmetastatic rectal cancer patients. (B) Spearman Rho correlation analysis of CD200 and its receptor CD200R1. Correlation considered as significant at the 0.01 and 0.05 levels (2- tailed).
