# Supplementary material for: Interaction of CD200 Overexpression on Tumor Cells with CD200R1 Overexpression on Stromal Cells: An Escape from the Host Immune Response in Rectal Cancer Patients
Source: J Oncol. 2019 Jan 21;2019:5689464. doi: 10.1155/2019/5689464 (PMC6360612; doi:10.1155/2019/5689464)
Supplement: Supplementary Materials — Supplementary Table 1. Immunohistochemical scoring of CD200 in rectal cancer tissues compared to the benign epithelium (p=0.001). Supplementary Table 2. Comparative analysis of CD200R1 expression in cases with recurrence and without recurrence. Scoring was assessed on the stromal region of primary tumor samples. Supplementary Table 3. Comparative analysis of CD200R1 expression on stromal region of primary tumor sections. Cases compared between with or without lymph node metastasis. Supplementary Table 4. (A) The expression pattern of CD200 and CD200R1 in metastatic versus nonmetastatic rectal cancer patients. (B) Spearman Rho correlation analysis of CD200 and its receptor CD200R1. Correlation considered as significant at the 0.01 and 0.05 levels (2- tailed). [file 5689464.f1.pdf]

SUPPLEMENTARY TABLES AND LEGENDS

Supplementary Table 1. Immunohistochemical scoring of CD200 in rectal cancer tissues compared to the benign epithelium (p=0.001).

	**Rectal Cancer n = 140**	**Normal Mucosa n = 121**	**P Values**
**Range (mean)**	4 – 7 (6)	0 – 6 (3)	P = 0.001

Supplementary Table 2. Comparative analysis of CD200R1 expression in cases with recurrence and without recurrence. Scoring was assessed on the stromal region of primary tumor samples.

	**High Recurrence Risk Group n = 34**	**No recurrence Group n = 106**	**P Values**
**CD200R1 Scoring Range (mean)**	5 – 6 (5.5)	2 – 5 (4.0)	P = 0.012
**Relative Risk (95% Confidence)**	2.75 (1.50 – 4.75)	1.00	P = 0.001

Supplementary Table 3. Comparative analysis of CD200R1 expression on stromal region of primary tumor sections. Cases compared between with or without lymph node metastasis.

	**Metastasis Group n = 50**	**No Metastasis Group n = 90**	**P Values**
**CD200R1 Scoring Range (mean)**	6 – 7 (6.5)	3 – 5 (4.5)	P = 0.001
**Relative Risk (95% Confidence)**	3.07 (1.10 – 5.75)	1.00	P=0.001

Supplementary Table 4. (A) The expression pattern of CD200 and CD200R1 in metastatic versus non-metastatic rectal cancer patients. **(B)** Spearman rho correlation analysis of CD200 and its receptor CD200R1. Correlation considered as significant at the 0.01 and 0.05 levels (2-tailed).

A

	**Cases**	**CD200 Score Mean (range)**	**CD200R1 Score Mean (range)**	
			**Tumoral**	**Stromal**
**Metastatic**				
**RT Group**	20	6 (6-7)	4 (3 – 6)	6.5 (6 – 7)
**Untreated**	30	6 (6-7)	4 (2 – 6)	6 (6 – 7)
**Non-metastatic**				
**RT Group**	41	4 (4-6)	3.5 (3 – 5)	4 (3 – 6)
**Untreated**	49	4 (4-6)	3.5 (2 – 5)	4 (2 - 6)

B

**Spearman's Rho**	**CD200**	**CD200R1 Tumoral**	**CD200R1 Stromal**
**CD200**			
Correlation Coefficient	1.000	.314	.155
sig. (2-tailed)	.	.174	.607
**CD200R1 Tumoral**			
Correlation Coefficient	.314	1.000	.216
Sig. (2-tailed)	.174	.	.268
**CD200R1 Stromal**			
Correlation Coefficient	.155	.216	1.000
sig. (2-tailed)	.607	.268	.
